# What comes first: Heart rate variability changes or insomnia? A causal investigation using Mendelian randomization

**DOI:** 10.1016/j.ijchp.2025.100656

**Published:** 2025-12-12

**Authors:** Bing Yan, Xin Cui, Lei Chen, Xiangzhen Miao, Hailin Jiang, Yu Wang, Fuchun Wang

**Affiliations:** a Institute of Basic Research in Clinical Medicine, China Academy of Chinese Medical Sciences, Beijing, 100700, China; b School of Acupuncture-Moxibustion and Tuina, Changchun University of Chinese Medicine, Changchun, 130117, China; c School of Chinese Materia Medica, Beijing University of Chinese Medicine, Beijing 102488, China; d Graduate School, Changchun University of Chinese Medicine, Changchun 130117, China; e Institute of Acupuncture and Moxibustion, China Academy of Chinese Medical Sciences, Beijing 100700, China

**Keywords:** Heart rate variability, Insomnia, Mendelian randomization, Autonomic function, Sleep disorders

## Abstract

**Background:**

Heart rate variability (HRV), a vital indicator of autonomic nervous system function, is frequently altered in patients with insomnia. While substantial evidence suggests a link between HRV changes and insomnia, previous studies have been limited by small sample sizes and cross-sectional designs, making it impossible to determine whether HRV changes are a cause or consequence of insomnia. Understanding this causal relationship is crucial for developing targeted interventions for sleep disorders.

**Objective:**

To investigate the potential bidirectional causal relationship between HRV and insomnia using two-sample Mendelian randomization (MR) analysis.

**Method:**

We obtained genome-wide association study (GWAS) summary statistics for three HRV traits (pvRSA/HF, RMSSD, and SDNN) from IEU Open GWAS database and insomnia data from FinnGen database (5763 cases and 445,483 controls). Bidirectional MR analyses were performed using multiple methods including inverse variance weighted (IVW), MR-Egger, weighted median, and mode-based approaches. Sensitivity analyses were conducted to assess heterogeneity and horizontal pleiotropy.

**Results:**

MR analysis of HRV on insomnia revealed significant causal effects, specifically for pvRSA/HF (OR = 1.437, 95 % CI: 1.147–1.801, *p* = 0.002) and SDNN (OR = 1.910, 95 % CI: 1.092–3.342, *p* = 0.022). These associations remained robust across multiple MR methods and sensitivity analyses. In the reverse direction, MR analysis of insomnia on HRV found no significant causal effects on any HRV traits, suggesting unidirectional causality from HRV to insomnia.

**Conclusions:**

This study provides genetic evidence that altered HRV may causally increase insomnia risk, while insomnia does not appear to causally affect HRV. These findings suggest that regulating HRV might help alleviate insomnia symptoms and improve sleep quality, and highlight the potential of HRV as a therapeutic target in sleep disorders.

## Introduction

Sleep is a complex physiological process characterized by regular fluctuations in autonomic nervous activity, with alternating dominance of the sympathetic nervous system (SNS) and the parasympathetic nervous system (PNS). Disruptions in sleep result in predominance of SNS activity, while PNS activity diminishes, leading to characteristic changes in HRV (Tobaldini et al., 2013). In the context of modern, fast-paced lifestyles and increasing work-related stress, sleep disorders have become among the most commonly encountered conditions in clinical practice. Insomnia, the most prevalent of these disorders, is defined by persistent difficulties in falling asleep, staying asleep, or waking up too early (Perlis et al., 2022). Studies indicate that from 2017 to 2022, 29.8 % of the U.S. population experienced sleep disturbances, with approximately 27 % of the global population reporting similar issues (Di et al., 2022). As a result, insomnia has emerged as a significant global public health concern (Lim et al., 2023).

Research has demonstrated a strong link between insomnia and autonomic nervous system (ANS) dysfunction, with physiological manifestations including elevated body temperature, increased electrodermal activity, and reduced HRV (Calandra-Buonaura et al., 2016; Farina et al., 2014). Moreover, insomnia may considerably increase the risk of cardiovascular diseases via mechanisms involving reduced HRV and elevated heart rate. These physiological changes underscore the profound impact of insomnia on overall health through ANS imbalance.

The intricate relationship between HRV and insomnia has garnered increasing attention. Observational studies consistently show a marked reduction in HRV among individuals with chronic insomnia, reflecting impaired autonomic nervous function. For instance, as early as 1998, Bonnet et al. (Bonnet et al., 1998) reported elevated low frequency (LF) and reduced high frequency (HF) components in the HRV of insomnia patients, indicating excessive SNS activation and diminished PNS function. Subsequent studies, such as those by Yang et al. (Yang et al., 2011), further demonstrated that reduced HF in insomnia patients reflects weakened vagal nerve function. Similarly, Hao et al. (2016) showed that HRV frequency-domain parameters (e.g., total power (TP), LF, HF, and the LF/HF ratio) are closely correlated with insomnia severity, suggesting that HRV may serve as a valuable indicator for assessing autonomic nervous function in patients with insomnia.

Conversely, alterations in HRV may precede the development of insomnia. Some studies suggest that specific HRV patterns could serve as early biomarkers for insomnia. For example, David Eddie et al. (Eddie et al., 2020) investigated HRV during wakefulness and sleep deprivation to explore the neurobiological basis of insomnia, examining HRV differences across various sleep stages. Their findings confirmed HRV as a potential biomarker for assessing neurobiological function. Reduced HRV may indicate early SNS overactivation, a state that increases sensitivity to stress or environmental changes, thus contributing to the onset of insomnia (Bonnet et al., 2010). SNS activation not only elevates heart rate and blood pressure but also disrupts sleep patterns by influencing emotional fluctuations and inertia (Owens et al., 2015; Wang et al., 2020). Therefore, reduced HRV functions as both an indicator of SNS overactivation and a potential mechanism underlying the development of insomnia.

The complexity of this causal relationship implies that autonomic nervous dysfunction may act as both a precursor and a consequence of insomnia. However, traditional observational studies face inherent limitations in establishing causality due to confounding factors and reverse causality. These challenges highlight significant gaps in our understanding of the temporal and causal relationships between HRV and insomnia, emphasizing the need for innovative research methodologies to address these issues.

Mendelian Randomization is an innovative research approach that uses genetic variations as instrumental variables (IVs) to examine causal relationships between exposures and outcomes. By minimizing confounding bias and reverse causality, MR offers stronger evidence for causal inference (Lawlor et al., 2008). Recently, MR has been successfully applied to investigate relationships between various health conditions, including sleep disorders and cardiovascular parameters (Yang et al., 2022, Yuan et al., 2021, Zeng et al., 2024, Zhang et al., 2024). However, no studies have yet specifically explored the causal directionality between HRV changes and insomnia.

This study aims to investigate the potential bidirectional causal relationship between changes in HRV and insomnia using two-sample MR analysis. Data on three HRV traits—the peak-valley respiratory sinus arrhythmia or high-frequency power (pvRSA/HF), the root mean square of successive RR interval differences (RMSSD), and the standard deviation of normal-to-normal intervals (SDNN)—were obtained from the IEU Open GWAS database, while insomnia-related data were sourced from the FinnGen database. This approach addresses the inherent limitations of traditional observational studies and provides genetic evidence to clarify the causal relationship between HRV and insomnia. Understanding this causal relationship is crucial for both clinical practice and public health. On one hand, dynamic monitoring of HRV could help identify individuals at high risk for insomnia, enabling timely intervention. On the other hand, effective treatment of insomnia may improve HRV, thereby enhancing autonomic nervous system regulation. Furthermore, these findings may contribute to a deeper understanding of the complex relationship between sleep disorders and cardiovascular health, offering a solid scientific foundation for the development of more effective prevention and treatment strategies for both conditions.

## Materials and methods

### Study design

**Fig. 1:**
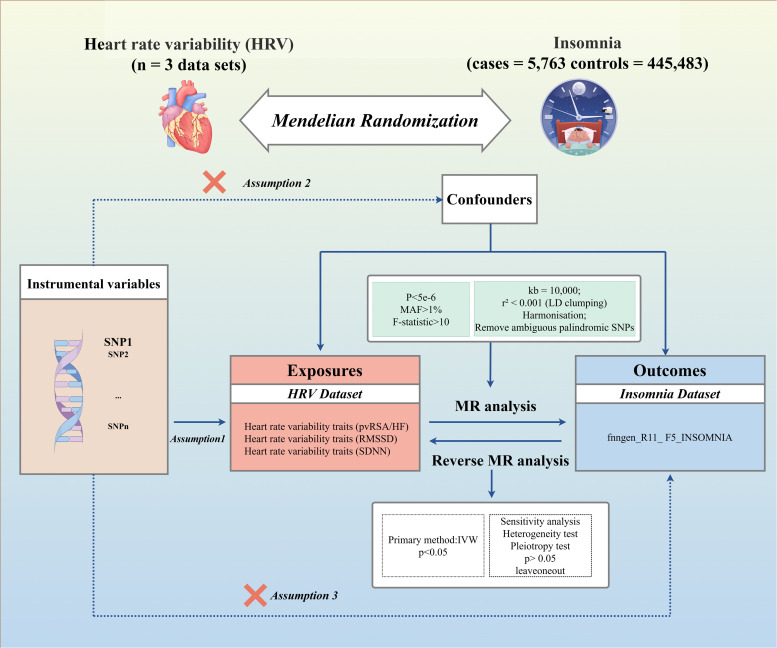
Overall design diagram for this study. IVW, inverse variance weighted; MR, mendelian randomization; pvRSA/HF, the peak-valley respiratory sinus arrhythmia or high-frequency power; RMSSD, the root mean square of successive RR interval differences; SDNN, the standard deviation of normal-to-normal intervals; SNP, single nucleotide polymorphism; HRV, heart rate variability.

Fig. 1 presents a schematic diagram of the study design. We employed a two-sample MR approach to examine the causal relationship between HRV and insomnia. To ensure the robustness of our results, we considered three key assumptions in the MR analysis (Davies et al., 2018). First, the genetic variants used as IVs must be robustly associated with the exposure of interest (HRV traits or insomnia).

Second, the IVs must be independent of any confounding factors that are related to both the exposure and the outcome. Third, the IVs should influence the outcome only through the exposure and not via alternative biological pathways (i.e. the exclusion restriction assumption). To enhance the transparency and rigor of this observational study, we adhered to the Strengthening the Reporting of Observational Studies in Epidemiology using MR (STROBE-MR) checklist (Appendix 1) (Skrivankova et al., 2021).

### Data sources and instruments

The genetic data used in this study were obtained exclusively from publicly available databases to ensure access to reliable datasets related to exposures and outcomes. The databases included the IEU Open GWAS (https://gwas.mrcieu.ac.uk/) (Hemani et al., 2018) and FinnGen (https://www.finngen.fi/en) (Kurki et al., 2023). Since all data were anonymized using sample identifiers, researchers could not access participants' personal identifying information, and no additional ethical approval was required.

To ensure scientific rigor and consistency, this study restricted the genetic background of participants to individuals of European ancestry. This restriction was necessary because data from other ancestral groups may not be generalizable to the studied traits. Additionally, differences in genetic population structure could introduce confounding factors, potentially affecting the accuracy of the analysis results. A bidirectional exposure-outcome framework was employed, with HRV-related indicators serving as exposure variables and insomnia as the outcome. This framework allowed the evaluation of both the potential causal effect of HRV on insomnia and the reverse causal effect of insomnia on HRV.

To comprehensively investigate HRV-related traits, three datasets from the IEU Open GWAS database were selected as exposure variables. Specifically, the following HRV traits were analyzed (Nolte et al., 2017): the SDNN dataset (GWAS ID: ebi-a-GCST004734), which included 27,850 individuals of European ancestry and 2549,728 single nucleotide polymorphisms (SNPs); the RMSSD dataset (GWAS ID: ebi-a-GCST004733), which included 26,523 individuals of European ancestry and 2528,639 SNPs; and the pvRSA/HF dataset (GWAS ID: ebi-a-GCST004732), which included 24,088 individuals of European ancestry and 2526,821 SNPs.

The insomnia-related GWAS data were sourced from the FinnGen database (https://r12.finngen.fi/pheno/F5_INSOMNIA). Specifically, the finngen_R11_F5_INSOMNIA dataset was utilized, with insomnia diagnosed according to the criteria established in the International Classification of Sleep Disorders, 3rd edition (ICSD-3) (American Academy of Sleep Medicine, 2023). This dataset comprised 451,246 individuals of European ancestry, including 5763 insomnia cases and 445,483 controls, with 21,306,764 genetic variants available.

### Selection of instrumental variables

#### HRV exposure data

To identify SNPs strongly associated with HRV, we set a screening threshold of *p* < 5 × 10⁻⁶ (Yu et al., 2023). To avoid linkage disequilibrium (LD), we used the European LD reference panel for common SNPs implemented in the “ieugwasr” package in R (version 4.5.2; https://github.com/MRCIEU/ieugwasr). SNPs in high LD (r² > 0.001 within a 10,000-kb window) were excluded (Cui et al., 2024).

We then used the `get_r_from_bsen` function in “ieugwasr” to approximate the F-statistic for multiple SNPs, calculated as follows:

*R* = get_r_from_bsen(beta, se, samplesize);

*F* = (samplesize − 2) × (R² / (1 − *R*²)).

Here, beta, se and samplesize represent the effect size, standard error and sample size, respectively. Instrument strength was assessed using the F-statistic, and variants with *F* > 10 were retained to mitigate weak-instrument bias. Subsequently, we excluded SNPs with a minor allele frequency (MAF) < 1 % and palindromic SNPs with ambiguous strand information. The remaining SNPs were used as IVs for the forward MR analyses.

#### Insomnia outcome data

For the forward MR analyses (HRV traits as exposures and insomnia as the outcome), summary-level associations of the selected HRV instruments with insomnia were extracted from the finngen_R11_F5_INSOMNIA GWAS. Effect alleles were aligned between the HRV and insomnia datasets, and palindromic SNPs with ambiguous allele frequencies were removed to ensure consistent orientation. No additional filtering of instruments was undertaken on the basis of their association p-values in the insomnia GWAS.

#### Insomnia exposure data

For the insomnia GWAS data, SNPs significantly associated with insomnia were identified using a threshold of *p* < 1 × 10⁻⁵ (Huang et al., 2024). To account for LD effects, SNPs with r² > 0.001 within a 10,000-kb window were excluded using the European LD reference panel in the “ieugwasr” package. The `get_r_from_bsen` function was applied to approximate the F-statistic for multiple SNPs, ensuring F-statistic values greater than 10 to minimize weak instrument bias. Finally, SNPs with MAF < 1 % and palindromic SNPs were excluded. The remaining SNPs were used as IVs for the subsequent MR analyses.

#### HRV outcome data

For the reverse MR analyses, summary-level associations for the insomnia instruments with each HRV trait were obtained from the Nolte et al. GWAS datasets. Harmonization and quality-control procedures between the insomnia and HRV GWAS datasets were identical to those described in Section 2.3.2, including alignment of effect alleles and removal of palindromic SNPs with ambiguous strand information. Consistent with the forward analyses, SNPs were not excluded on the basis of their association p-values in the HRV outcome GWAS.

### MR analysis

We conducted bidirectional two-sample MR analyses in R (version 4.5.2) using the “TwoSampleMR” package (version 0.6.24; https://mrcieu.github.io/TwoSampleMR/index.html) (Yang et al., 2023) to evaluate the causal relationship between HRV and insomnia. Five MR methods were applied: MR-Egger (Bowden et al., 2015), IVW method (Pierce et al., 2013), Weighted Median (Bowden et al., 2016), Simple Mode (Du et al., 2023), and Weighted Mode (Wang et al., 2024).

To assess heterogeneity in the model, Cochran’s Q statistic was calculated using the MR-Egger and IVW methods. This was implemented through the mr_heterogeneity function in the Two Sample MR package. The formula is as follows:

$$Q=\sum_{j}w_{j}{\left({\hat{\beta }}_{j}-\hat{\beta }\right)}^{2}$$

Where ${\hat{\beta }}_{j}$ is the estimated effect of the j^th^ IV, $w_{j}$ is the corresponding weight, and $\hat{\beta }$ is the pooled effect estimate derived from either the IVW or MR-Egger method. A Q statistic P-value greater than 0.05 indicates no significant heterogeneity in the model (Li et al., 2024). The reliability of the results was further assessed by examining the consistency across multiple MR methods.

Horizontal pleiotropy was evaluated using MR-Egger regression. A significant deviation of the MR-Egger intercept from zero indicates the presence of horizontal pleiotropy, which occurs when IVs directly influence the outcome through non-exposure pathways, thus violating MR assumptions. A P-value greater than 0.05 suggests the absence of significant horizontal pleiotropy (Cao et al., 2022).

To further assess the impact of pleiotropic outliers, we applied the MR pleiotropy residual sum and outlier (MR-PRESSO) method (Chen et al., 2022). This analysis was performed with 1000 iterations to identify and exclude potential outliers, thereby reducing bias from pleiotropy.

For better visualization of the analysis results, scatter plots and funnel plots were generated. The scatter plots display the distribution of effect estimates for the IVs, while the funnel plots illustrate the results of pleiotropy tests, providing a more intuitive understanding of the data.

## Results

### Selection of IVs for MR analysis

Applying the instrumental-variable selection criteria described in Section 2.3, we retained 10 SNPs as instruments for pvRSA/HF, 17 SNPs for RMSSD and 17 SNPs for SDNN after harmonisation with the insomnia GWAS. Detailed information on these SNPs is provided in Appendix 2.

### MR results for HRV and insomnia

As outlined in the Methods, the three HRV-related GWAS datasets were used as exposure data, with insomnia as the outcome in the forward MR analyses. The primary IVW estimates indicated significant causal associations of pvRSA/HF and SDNN with insomnia, whereas the evidence for RMSSD was weaker.

As shown in Table 1, a genetically predicted increase in pvRSA/HF was associated with a higher risk of insomnia (IVW OR = 1.437, 95 % CI = 1.147–1.801, *p* = 0.002). Similarly, higher SDNN was associated with increased odds of insomnia (IVW OR = 1.91, 95 % CI = 1.09–3.34, *p* = 0.023). For RMSSD, the point estimate was directionally consistent but did not reach statistical significance (IVW OR = 1.268, 95 % CI = 0.848–1.896, *p* = 0.247).

**Table 1:** MR analysis results of HRV and insomnia.

Outcome	Exposure	Method	Nsnp	Pval	OR	95 %CI
Insomnia	HRV traits (pvRSA/HF)	IVW	10	0.002	1.437	1.147–1.801
Insomnia	HRV traits (RMSSD)	IVW	17	0.247	1.268	0.848–1.896
Insomnia	HRV traits (SDNN)	IVW	17	0.022	1.910	1.092–3.342

Abbreviations: HRV, Heart rate variability; pvRSA/HF, Peak-valley respiratory sinus arrhythmia or high-frequency power SDNN, Standard deviation of normal-to-normal intervals; RMSSD, Root mean square of successive RR interval differences; IVW, inverse variance weighted.

Scatter plots illustrating the MR estimates across the five methods (IVW, MR-Egger, weighted median, simple mode and weighted mode) are provided in Supplementary Figure S1 and show that the causal estimates for pvRSA/HF and SDNN were consistently positive, supporting the finding that increased HRV—particularly pvRSA/HF and SDNN—is associated with a higher risk of insomnia.

### Sensitivity analysis of MR results for HRV and insomnia

We conducted a series of sensitivity analyses to assess the robustness of the forward MR findings, including heterogeneity tests, pleiotropy tests and leave-one-out analyses. As shown in the leave-one-out plots (Supplementary Figure S2), sequential exclusion of individual SNPs did not materially change the effect estimates for pvRSA/HF or SDNN on insomnia; all estimates remained on the same side of the null, and the overall MR results exhibited minimal fluctuation.

Heterogeneity was evaluated using Cochran’s Q statistic under the IVW and MR-Egger models, and horizontal pleiotropy was assessed using the MR-Egger intercept test and the MR-PRESSO global test (Table 2). For pvRSA/HF, neither the Q statistics nor the pleiotropy tests provided evidence of major violations of the MR assumptions (MR-Egger Q_*p* = 0.18, IVW Q_*p* = 0.14, Egger intercept *p* = 0.25, MR-PRESSO global test *p* = 0.15), supporting the robustness of the positive association with insomnia. For RMSSD, the IVW heterogeneity test (Q_*p* = 0.02), the MR-Egger intercept (*p* = 0.02) and the MR-PRESSO global test (*p* = 0.01) indicated some heterogeneity and directional pleiotropy, which is consistent with the absence of a robust causal association for this trait. For SDNN, there was modest heterogeneity and evidence of global pleiotropy (MR-Egger Q_*p* = 0.05, IVW Q_*p* = 0.03, MR-PRESSO *p* = 0.04), although the MR-Egger intercept did not suggest directional pleiotropy (*p* = 0.21). Overall, these sensitivity analyses suggest that the causal link between pvRSA/HF and insomnia is relatively robust, while the SDNN estimates should be interpreted with caution and the RMSSD findings do not support a clear causal effect.

**Table 2:** Heterogeneity and pleiotropy in the MR analysis of HRV and insomnia.

Heterogeneity test						Pleiotropy test		
Outcome	Exposure	Method	Q	Q_df	Q_pval	Egger_intercept	SE	Pval
Insomnia	HRV traits (pvRSA/HF)	MR Egger	11.386	8	0.181	−0.0219	0.018	0.253
		IVW	13.546	9	0.139			
		MR PRESSO						0.154
Insomnia	HRV traits (RMSSD)	MR Egger	20.821	15	0.143	−0.0437	0.017	0.019
		IVW	30.352	16	0.016			
		MR PRESSO						0.013
Insomnia	HRV traits (SDNN)	MR Egger	23.887	14	0.047	−0.0385	0.029	0.210
		I IVW	26.833	15	0.030			
		MR PRESSO						0.035

**Abbreviations:** HRV, Heart rate variability; pvRSA/HF, Peak-valley respiratory sinus arrhythmia or high-frequency power SDNN, Standard deviation of normal-to-normal intervals; RMSSD, Root mean square of successive RR interval differences; IVW, inverse variance weighted; MR, mendelian randomization; PRESSO, pleiotropy residual sum and outlier.

### Selection of IVs for reverse MR analysis

Using the same instrumental-variable selection criteria for insomnia as the exposure (Section 2.3), 9 independent SNPs were retained as instruments for each reverse MR analysis with pvRSA/HF, RMSSD and SDNN as outcomes. Detailed information on these instruments is provided in Appendix 3.

### Reverse MR analysis of insomnia and HRV

We next performed reverse MR analyses to evaluate the potential causal effect of genetic liability to insomnia on HRV traits. As shown in Table 3, none of the IVW estimates provided evidence that insomnia causally influences pvRSA/HF, RMSSD or SDNN; all odds ratios were very close to 1.00 and their 95 % confidence intervals included the null. Scatter plots for the reverse MR analyses are presented in Supplementary Figure S3 and show that the point estimates from the five MR methods clustered around the null for all three HRV traits, with wide and overlapping confidence intervals. Taken together, these findings indicate a lack of robust evidence for a reverse causal effect of insomnia on HRV.

**Table 3:** MR analysis results of insomnia and HRV.

Outcome	Exposure	Method	Nsnp	Pval	OR	95 %CI
HRV traits (pvRSA/HF)	Insomnia	IVW	9	0.862	1.007	0.934–1.085
HRV traits (RMSSD)	Insomnia	IVW	9	0.764	1.006	0.970–1.043
HRV traits (SDNN)	Insomnia	IVW	9	0.392	1.011	0.986–1.038

**Abbreviations:** HRV, Heart rate variability; pvRSA/HF, Peak-valley respiratory sinus arrhythmia or high-frequency power SDNN, Standard deviation of normal-to-normal intervals; RMSSD, Root mean square of successive RR interval differences; IVW, inverse variance weighted.

### Sensitivity analysis of insomnia on HRV

Sensitivity analyses for the reverse MR models included heterogeneity tests, pleiotropy tests and leave-one-out analyses. As illustrated in the leave-one-out and funnel plots (Supplementary Figure S4), sequential exclusion of individual SNPs did not materially change the IVW estimates for any HRV trait; the effect sizes remained close to the null and showed minimal fluctuation, indicating that no single variant was driving the overall results. The funnel plots also appeared approximately symmetrical, further suggesting the absence of substantial small-study or directional bias.

Heterogeneity was assessed using Cochran’s Q statistic under the IVW and MR-Egger models, and horizontal pleiotropy was evaluated using the MR-Egger intercept and the MR-PRESSO global test (Table 4). For all three HRV traits, the Q statistics did not indicate significant heterogeneity (all Q_*p* ≥ 0.16). The MR-PRESSO global tests similarly did not detect any influential outliers (global test *p* ≥ 0.16). The MR-Egger intercept suggested a modest degree of directional pleiotropy for pvRSA/HF (intercept −0.0357, *p* = 0.026), whereas no evidence of pleiotropy was observed for RMSSD or SDNN (intercept *p* ≥ 0.087). Taken together with the IVW estimates, which were very close to 1.00 and had 95 % confidence intervals spanning the null for all traits, these findings indicate that genetic liability to insomnia does not exert a detectable causal effect on HRV, and that any residual pleiotropy is unlikely to materially influence this conclusion.

**Table 4:** Heterogeneity and pleiotropy in the MR analysis of insomnia and HRV.

Heterogeneity test						Pleiotropy test		
Outcome	Exposure	Method	Q	Q_df	Q_pval	Egger_intercept	SE	Pval
HRV traits (pvRSA/HF)	Insomnia	MR Egger	3.820	7	0.800	−0.0357	0.013	0.026
		IVW	11.758	8	0.162			
		MR PRESSO						0.164
HRV traits (RMSSD)	Insomnia	MR Egger	2.559	7	0.923	−0.0123	0.006	0.087
		IVW	6.532	8	0.588			
		MR PRESSO						0.576
HRV traits (SDNN)	Insomnia	MR Egger	4.534	7	0.717	−0.0034	0.004	0.472
		IVW	5.113	8	0.745			
		MR PRESSO						0.714

**Abbreviations:** HRV, Heart rate variability; pvRSA/HF, Peak-valley respiratory sinus arrhythmia or high-frequency power SDNN, Standard deviation of normal-to-normal intervals; RMSSD, Root mean square of successive RR interval differences; IVW, inverse variance weighted; MR, mendelian randomization; PRESSO, pleiotropy residual sum and outlier.

## Discussion

In this study, we utilized the MR method to examine the causal relationship between HRV and insomnia. Our findings revealed a significant causal link, with certain HRV characteristics increasing the risk of insomnia. To enhance our understanding, we also performed a reverse MR analysis to investigate the potential impact of insomnia on HRV. Overall, the sensitivity analyses suggested limited heterogeneity and, apart from a modest Egger intercept for pvRSA/HF, no strong evidence of directional pleiotropy, making major bias unlikely. However, the *p*-value derived from the IVW method, a key assessment tool, was not statistically significant, implying a lack of substantial evidence to support the hypothesis that insomnia affects HRV in a reverse causal manner. These findings suggest that HRV could be a potential risk factor for insomnia, with higher HRV associated with an increased risk of insomnia development. Conversely, the impact of insomnia on HRV appears negligible, suggesting that insomnia may be a consequence of altered HRV rather than a causative factor. This insight is valuable for understanding the etiology of insomnia and may prompt consideration of HRV management in insomnia interventions.

Studies investigating the impact of insomnia on HRV, particularly from small-sample clinical trials and animal experiments, often yield contradictory results. These inconsistencies could stem from factors such as small sample sizes, specific control conditions, or limitations in experimental settings. Such studies are typically conducted in controlled laboratory environments, which may not fully replicate the causal relationships observed in natural settings. For example, small sample sizes can limit statistical power, making it difficult to detect subtle but significant effects (Žunkovič et al., 2023). Furthermore, the controlled conditions in experimental settings may not reflect real-world environments, which can affect the generalizability of the findings.

While laboratory environments can influence HRV measurements, MR studies are based on large-scale population data derived from natural settings, providing broader applicability. Factors such as noise, temperature, and lighting in laboratory conditions may interfere with participants' physiological states, thus influencing HRV measurements (Schumann et al., 2021). In contrast, MR studies use genetic variants as IVs, enabling the assessment of long-term causal relationships in more natural environments and reducing the potential interference of laboratory conditions on the results (Burgess et al., 2021). Future research could integrate short-term HRV changes observed in experimental settings with long-term changes in natural conditions to develop a more comprehensive understanding of the relationship between insomnia and HRV.

### Heart rate variability and the autonomic nervous system

Heart rate variability serves as a non-invasive marker of ANS activity, reflecting the balance between the SNS and PNS (Chalmers et al., 2022). In individuals with insomnia, HRV metrics—such as time-domain indicators (e.g., SDNN, RMSSD) and frequency-domain indicators (e.g., HF)—typically show a downward trend, suggesting a disrupted ANS balance. This disruption is characterized by diminished PNS regulation and heightened SNS excitability (Chalmers et al., 2022). The excessive activation of the SNS can compromise sleep quality, thereby increasing the risk of insomnia. According to the hyperarousal hypothesis of insomnia, individuals with insomnia are in a state of chronic physiological and psychological hyperarousal, which is linked to overactivation of the SNS (Chalmers et al., 2022). For example, studies have shown that individuals with insomnia experience sustained sympathetic hyperactivation during the night. This overactivation may interfere with normal sleep processes by elevating physiological indicators such as heart rate and blood pressure, leading to symptoms like difficulty falling asleep and maintaining sleep (Chalmers et al., 2022). Therefore, abnormal HRV may serve as a physiological mechanism underlying insomnia, suggesting that regulating ANS function, particularly by inhibiting excessive SNS activation, may help alleviate insomnia symptoms.

### The interplay of stress, inflammation, and sleep

Psychological stress and inflammatory responses are key factors that influence both HRV and sleep, thereby contributing to insomnia. Psychological stress can activate the hypothalamic–pituitary–adrenal (HPA) axis and the sympathetic nervous system, leading to elevated levels of stress hormones such as cortisol and adrenaline. These hormones not only reduce HRV (Sugama & Kakinuma, 2020), but also disrupt sleep architecture and quality, thereby increasing the risk of insomnia (Dalfsen & Markus, 2018). For example, HPA axis activation in response to stress has been associated with sleep disturbances in individuals with insomnia (Dalfsen & Markus, 2018).

Inflammatory responses are also closely linked to both HRV and sleep (Koreki et al., 2024). Chronic inflammation can lead to elevated levels of pro-inflammatory cytokines (such as IL-6 and TNF-α), which may lower HRV and affect sleep-regulatory centres, ultimately impairing sleep quality. Observational studies have reported higher levels of inflammatory markers in people with insomnia, likely reflecting the combined impact of psychological stress and low-grade inflammation (Koreki et al., 2024). In line with this framework, HRV has been proposed as an integrated marker of stress- and inflammation-related autonomic imbalance, which could in turn increase insomnia risk.

In our study, we additionally performed exploratory MR analyses using CRP and IL-6 as prototypical systemic inflammatory markers, and found no robust causal effects of genetically predicted pvRSA/HF and SDNN on these markers (all IVW p-values > 0.20; Supplementary Table S1). These null results should be interpreted cautiously, as the number of available instruments was limited and we did not systematically evaluate a broader panel of stress- and inflammation-related traits (e.g. other cytokines or neuroendocrine markers such as cortisol). Nevertheless, they suggest that the HRV–insomnia association observed here is unlikely to be mediated solely through CRP- or IL-6–related systemic inflammation. Taken together, the current evidence is consistent with a model in which HRV may reflect the complex interplay between stress, inflammation and autonomic regulation, and interventions that reduce psychological stress and modulate inflammatory activity may help to improve HRV and alleviate insomnia symptoms.

### Interpretation of reverse MR analysis results

Firstly, we explore potential explanations for the influence of insomnia on HRV. Although the reverse analysis did not find a significant impact of insomnia on HRV, it is plausible that insomnia primarily affects short-term heart rate fluctuations. Mendelian randomization methods are more suited for inferring long-term causal relationships, as they rely on genetic variants that reflect chronic and long-term associations between insomnia and HRV (Tian et al., 2021). The ANS of individuals with insomnia is often imbalanced, leading to reduced HRV. However, these short-term physiological changes may not be evident in long-term genetic analyses (Ma et al., 2024).

Next, we address the disparities between short-term and long-term impacts. The short-term effects of insomnia on HRV, observed in small-sample experiments, may not be detectable in long-term genetic analyses. Short-term experiments typically examine the effects of insomnia over a brief period, such as acute sleep deprivation, which can lead to increased SNS activity and a reduction in specific HRV indicators (Ma et al., 2024). These immediate effects are readily detectable in laboratory settings. In contrast, MR analysis provides specificity for long-term causal inference, emphasizing chronic risk patterns (Tian et al., 2021).

We also consider the complementary role of reverse MR analysis in relation to short-term experimental findings. The absence of a significant long-term impact of insomnia on HRV in reverse MR analysis does not imply that insomnia does not affect HRV in the short term. Rather, it suggests that insomnia may not have a substantial direct effect in the long-term genetic context (Tian et al., 2021). Short-term experiments reveal the immediate physiological stress response caused by insomnia, while reverse MR analysis excludes significant causal relationships of insomnia on HRV in the long-term genetic context (Ma et al., 2024). This complementarity suggests that long-term changes in HRV are more likely influenced by physiological foundations or chronic health factors, rather than by insomnia alone. Thus, reverse MR analysis offers a stable perspective on the long-term relationship between insomnia and HRV, while short-term experiments help elucidate the immediate physiological changes induced by insomnia.

### Methodological advantages and limitations

The MR approach has important strengths for investigating the relationship between HRV and insomnia. By using germline genetic variants as IVs, MR can largely minimise confounding and reverse causation, thereby improving the reliability of causal inference compared with conventional observational designs (Relton & Davey, 2015). In our study, multiple independent variants were combined in the IVW framework to obtain a weighted causal estimate, and a series of sensitivity analyses (MR-Egger, weighted median, MR-PRESSO, Cochran’s Q and leave-one-out analyses) were used to check for heterogeneity and horizontal pleiotropy. Compared with small clinical trials or animal experiments, MR is particularly suited to assessing long-term causal effects at the population level, as it is not constrained by short follow-up, small sample sizes or imperfect randomisation (Hirst et al., 2014).

Several limitations should also be acknowledged. First, all GWAS datasets were restricted to individuals of European ancestry, which reduces population stratification but limits the generalisability of our findings to other ancestries. Second, our insomnia outcome was obtained from the FinnGen GWAS, which offers a clinically ascertained registry-based phenotype and fully open summary statistics but has a smaller sample size than the largest available insomnia meta-analysis (Watanabe et al., 2022). In addition, the insomnia phenotype was modelled as a single binary endpoint and did not distinguish specific clinical subtypes (e.g. sleep-onset, sleep-maintenance or early-morning awakening insomnia), so we could not assess potential differences in causal effects across insomnia subphenotypes. This trade-off between phenotype definition, data accessibility and statistical power may have reduced our ability to detect more modest effects, especially in the reverse MR analyses. Leveraging larger meta-analytic GWAS in future MR studies will be instrumental in replicating and refining these causal estimates. Third, as with any MR study, residual horizontal pleiotropy and violations of the exclusion-restriction assumption cannot be completely excluded despite extensive sensitivity analyses (Relton & Davey, 2015). Finally, MR estimates reflect the lifelong effects of genetically influenced differences rather than short-term changes induced by specific interventions; therefore, complementary evidence from clinical trials and mechanistic animal studies remains essential to validate and extend our findings on the complex relationship between HRV and insomnia.

### Future directions

The causal relationship between HRV and insomnia holds significant potential for clinical application. HRV monitoring is expected to evolve into an effective preventive tool, enabling real-time detection and early warning of insomnia risk through wearable devices. While certain medications have shown promising efficacy in treating insomnia, challenges such as adverse effects and limited safety data remain De Crescenzo et al. (2022). Consequently, non-pharmacological therapies have garnered increasing attention, particularly interventions targeting HRV, such as cognitive behavioral therapy for insomnia (CBT-I) and physical therapies (Morin & Buysse, 2024), offering novel strategies for insomnia management.

Through a comprehensive review of the literature, we summarize the effects of non-pharmacological treatments on HRV metrics in recent years. These therapies primarily include biofeedback technology (Yen et al., 2023; de Zambotti et al., 2019; Zucker et al., 2009; Lin et al., 2019; Herhaus et al., 2022), CBT-I (Amra et al., 2023; Jarrin et al., 2016), acupuncture therapy (Chen et al., 2024; Lee et al., 2009; Fasmer et al., 2012), and transcutaneous auricular vagus nerve stimulation (taVNS) (Wu et al., 2021; Zhao et al., 2022) (Table 5). The mechanisms of these therapies are thought to involve the enhancement of PNS activity to restore ANS balance, leading to significant improvements in HRV metrics such as SDNN, LF, and HF. Improvements in HRV reflect a more balanced and stable ANS, thereby enhancing sleep quality in individuals with insomnia.

**Table 5:** Relationship between HRV and insomnia and non-pharmacological treatment.

Non-pharmacological Therapies Category	Changes in HRV Indicators After Treatment of Insomnia	Improved Sleep Indicators After Treatment	References
Biofeedback (HRVB/MHRVB/IARB/RSAB)	HF(↑); LF(↑); mHR(↓); SDNN (↑); TP (↑)	PSQI(TS, subjective sleep quality score); PSG(awakening index;sleep stage transitions); ISI(TS); PSQI (TS); Pre-Sleep Arousal Scale (TS, cognitive arousal score)	Yen et al., 2023; de Zambotti et al., 2019; Zucker et al., 2009; Lin et al., 2019; Herhaus et al., 2022
CBT-I	HF (↑); LF/HF (↑/↓); HF (↑)	ISI(TS); PSG(SOL,WASO,TST,SE); Sleep diary(SE, TST, SOL)	Amra et al., 2023; Jarrin et al., 2016;
Acupuncture/Intradermal acupuncture	HF (↑/↓);LF(↓); LF/HF(↓)	ISI (TS); AIS (TS)	Chen et al., 2024; Lee et al., 2009; Fasmer et al., 2012;
taVNS/Auricular acupressure	HF(↑);PNN50 (↑); LF(↑/↓); LF/HF(↑); SDNN(↑); NNMean(↑); RMSSD(↑)	PSQI(TS); PSG(SL, WASO, SE, N3, TST)	Wu et al., 2021; Zhao et al., 2022; Kung et al., 2011;
Exercise training	HF(↓); LF/HF(↓)	PSQI(TS, subjective Sleep duration, Sleep disturbances, SOL, Daytime dysfunction, SE, Subjective sleep quality, Use of sleeping medication, Self-reported sleep duration and Self-reported SOL); Actigraph(SOL, SE)	Tseng et al., 2020;
Binaural beat music therapy	SDNN(↑); LF(↓); mHR(↑)	PSQI(TS, subjective sleep quality score, and habitual SE)	2024; ;
Lavender aromatherapy	SDNN(↑); RMSSD(↑); HF(↑)	PSQI(TS)	Chien et al., 2012;
Closed-loop allostatic neurotechnology	SDNN(↑); RMSSD(↑); HF(↑); LF(↑); TP(↑)	ISI(TS)	Tegeler et al., 2017;
Vestibular stimulation	LF(↑)	the latency from bedtime to persistent sleep	Campana et al., 2011;
HIRREM	SDNN(↑); RMSSD(↑)	ISI(TS); Sleep diary(SE, TST, SOL)	Tegeler et al., 2020;

**Note:** ‘↓’ represents a decrease in HRV measurements in insomnia patients compared to treatment controls, while ‘↑’ represents an increase in HRV measurements in insomnia patients compared to treatment controls. ‘↑/↓’ indicates an increase or decrease in HRV measurements in insomnia patients relative to treatment controls.

**Abbreviations:** HRVB, Heart rate variability biofeedback; MHRVB, Mobile heart rate variability biofeedback; IARB, Immersive audio-visual respiratory biofeedback; RSAB, Respiratory sinus arrhythmia biofeedback; CBT-I, Cognitive behavioral therapy for insomnia; taVNS, Transcutaneous auricular vagus nerve stimulation; HIRREM, High‐resolution, relational, resonance‐based, electroencephalic mirroring; HF, High frequency; LF, Low frequency; LF/HF, Ratio of low-to-high frequency power; mHR, Mean heart rate; SDNN, Standard deviation of normal-to-normal intervals; TP, Total power; PNN50, Percentage of successive RR intervals differing by >50 ms; NNMean, Mean of Normal-to-Normal Intervals; RMSSD, Root mean square of successive RR interval differences; PSQI, Pittsburgh Sleep Quality Index; TS, The total score; PSG, Polysomnographic sleep assessment; ISI, Insomnia Severity Index; SOL, Sleep onset latency; WASO, Wake after sleep onset; SE, Sleep efficiency; TST, Total sleep time; N3, Percentages of non-rapid eye movement period 3; AIS, Athens insomnia scale.

Future research should focus on validating the clinical efficacy of these non-pharmacological therapies and exploring their potential for insomnia prevention and management. Additionally, in-depth investigations are necessary to further our understanding of the relationship between insomnia and HRV. For example, by integrating MR with clinical trial designs, researchers can comprehensively evaluate the causal relationship between insomnia and HRV, considering both short-term and long-term effects. Expanding the research scope to include HRV indices beyond pvRSA/HF, HF, and SDNN could uncover novel mechanisms underlying their relationship. Furthermore, individualized analyses of specific populations are needed to examine the differential effects of HRV on insomnia across various groups and health conditions, thus improving the generalizability and clinical applicability of the findings. These directions will establish a robust scientific foundation for the precise prevention and personalized management of insomnia.

## Conclusion

Our results suggest no causal effect of insomnia on HRV, reinforcing the notion that HRV dysregulation may contribute to insomnia, rather than the reverse. These findings provide new insights into the etiology of insomnia and highlight the potential of HRV as a predictive factor for its prevention and management.

## Fundings

This work was supported by the Young Scientists Fund of the National Natural Science Foundation of China (No. 82004472), the National Key R&D Program of China (No. 2022YFC3500500, 2022YFC3500501), the Fundamental Research Funds for the Central Public Welfare Research Institutes (No. ZZ-2023006), and the Construction Project of High-Level Key Disciplines of Traditional Chinese Medicine of the State Administration of Traditional Chinese Medicine (No. zyyzdxk-2023221).

## Ethics

This study utilized anonymized data from the IEU Open GWAS (https://gwas.mrcieu.ac.uk/) and FinnGen (https://www.finngen.fi/en) databases. All data were anonymized using sample identifiers, ensuring that researchers could not access any personally identifiable information of participants. As a result, no additional ethical approval was required for this study.

## Declaration of generative AI and AI-assisted technologies in the writing process

During the preparation of this work, we used ChatGPT in order to improve the language and readability of the manuscript. After using this tool, we reviewed and edited the content as needed and take full responsibility for the content of the publication.

## Declaration of competing interest

The authors declare that they have no known competing financial interests or personal relationships that could have appeared to influence the work reported in this paper.

## Appendix Supplementary materials

Image, application 1

Image, application 2

Image, application 3

Image, application 4

Image, application 5

Image, application 6

Image, application 7

Image, application 8
