# Supplementary material for: What comes first: Heart rate variability changes or insomnia? A causal investigation using Mendelian randomization
Source: Int J Clin Health Psychol. 2025 Dec 12;25(4):100656. doi: 10.1016/j.ijchp.2025.100656 (PMC12765190; doi:10.1016/j.ijchp.2025.100656)
Supplement: Supplementary file 1 [file mmc1.docx]

**STROBE-MR checklist of recommended items to address in reports of Mendelian randomization studies**

| **Item No.** | **Section** | **Checklist item** | **Page No.** | **Relevant text from manuscript** |
| --- | --- | --- | --- | --- |
| 1 | **TITLE and ABSTRACT** | Indicate Mendelian randomization (MR) as the study’s design in the title and/or the abstract if that is a main purpose of the study | 1 | Title: “What Comes First: Heart Rate Variability Changes or Insomnia? A Causal Investigation Using Mendelian Randomization”; Abstract states “…using two-sample Mendelian randomization (MR) analysis.” |
|  | **INTRODUCTION** |  |  |  |
| 2 | **Background** | Explain the scientific background and rationale for the reported study. What is the exposure? Is a potential causal relationship between exposure and outcome plausible? Justify why MR is a helpful method to address the study question | 2–3 | 1^st^–7^th^ paragraphs of Introduction describe HRV, insomnia, their observational association and limitations of prior studies, and why MR is needed to clarify causality. |
| 3 | **Objectives** | State specific objectives clearly, including pre-specified causal hypotheses (if any). State that MR is a method that, under specific assumptions, intends to estimate causal effects | 3 | Last paragraph of Introduction (“This study aims to investigate the potential bidirectional causal relationship…”), explicitly stating bidirectional MR objectives. |
|  | **METHODS** |  |  |  |
| 4 | **Study design and data sources** | Present key elements of the study design early in the article. Consider including a table listing sources of data for all phases of the study. For each data source contributing to the analysis, describe the following: |  |  |
|  | a) | Setting: Describe the study design and the underlying population, if possible. Describe the setting, locations, and relevant dates, including periods of recruitment, exposure, follow-up, and data collection, when available. | 3–4 | 2.1 Study design describes bidirectional two-sample MR framework and use of summary-level GWAS data; 2.2 Data Sources and Instruments describes public databases (IEU Open GWAS, FinnGen) and European ancestry restriction. |
|  | b) | Participants: Give the eligibility criteria, and the sources and methods of selection of participants. Report the sample size, and whether any power or sample size calculations were carried out prior to the main analysis | 4 | 2.2 Data Sources and Instruments gives GWAS IDs, ancestry, and sample sizes for each HRV trait and the FinnGen insomnia dataset (5,763 cases, 445,483 controls). No separate power calculation performed. |
|  | c) | Describe measurement, quality control and selection of genetic variants | 4–5 | 2.3 Selection of Instrumental Variables (subsections 2.3.1–2.3.4) define p-value thresholds, LD clumping (r² < 0.001, 10,000-kb window), F-statistic > 10, MAF >1%, and removal of ambiguous palindromic SNPs for both HRV and insomnia instruments. |
|  | d) | For each exposure, outcome, and other relevant variables, describe methods of assessment and diagnostic criteria for diseases | 4 | 2.2 Data Sources and Instruments details HRV traits (pvRSA/HF, RMSSD, SDNN) from Nolte et al. GWAS and FinnGen insomnia phenotype defined according to ICSD-3 criteria. |
|  | e) | Provide details of ethics committee approval and participant informed consent, if relevant | 4 | 2.2 Data Sources and Instruments explains that only de-identified, publicly available GWAS summary data were used, therefore no additional ethics approval or consent were required. |
| 5 | **Assumptions** | Explicitly state the three core IV assumptions for the main analysis (relevance, independence and exclusion restriction) as well assumptions for any additional or sensitivity analysis | 3 | 2.1 Study design paragraph listing the three MR assumptions and explaining them, followed by statement that the study adheres to the STROBE-MR checklist. |
| 6 | **Statistical methods: main analysis** | Describe statistical methods and statistics used |  |  |
|  | a) | Describe how quantitative variables were handled in the analyses (i.e., scale, units, model) | 5-6 | 2.3 Selection of Instrumental Variables and 2.4 MR Analysis describe SNP effect estimates (beta, SE), approximation of F-statistics, and use of log-odds scale and ORs per genetically predicted increase in HRV / insomnia. |
|  | b) | Describe how genetic variants were handled in the analyses and, if applicable, how their weights were selected | 4-5 | 2.3 Selection of Instrumental Variables explains LD clumping, MAF thresholds, F-statistics, and use of SNP–exposure associations as weights in IVW and other MR methods. |
|  | c) | Describe the MR estimator (e.g. two-stage least squares, Wald ratio) and related statistics. Detail the included covariates and, in case of two-sample MR, whether the same covariate set was used for adjustment in the two samples | 5-6 | 2.4 MR Analysis lists IVW as primary method plus MR-Egger, weighted median, simple mode and weighted mode; notes use of two-sample MR with GWAS summary statistics that are adjusted for standard covariates by original consortia. |
|  | d) | Explain how missing data were addressed | 5 | 2.3 Selection of Instrumental Variables and 2.3.2–2.3.4 describe harmonisation between exposure and outcome GWAS and removal of SNPs with ambiguous alleles or incomplete information. |
|  | e) | If applicable, indicate how multiple testing was addressed | 5 | 2.3 Selection of Instrumental Variables notes p-value thresholds for SNP selection (p < 5×10⁻⁶ for HRV, p < 1×10⁻⁵ for insomnia) and use of conventional α = 0.05 for MR estimates; main focus on a limited set of pre-specified traits. |
| 7 | **Assessment of assumptions** | Describe any methods or prior knowledge used to assess the assumptions or justify their validity | 6-8 | 2.4 MR Analysis explains use of Cochran’s Q, MR-Egger intercept and MR-PRESSO to assess heterogeneity and horizontal pleiotropy; 3.3 and 3.6 report corresponding results for forward and reverse MR. |
| 8 | **Sensitivity analyses and additional analyses** | Describe any sensitivity analyses or additional analyses performed (e.g. comparison of effect estimates from different approaches, independent replication, bias analytic techniques, validation of instruments, simulations) | 6–8, 10 | 2.4 MR Analysis describes use of MR-Egger, weighted median, simple/weighted mode, Cochran’s Q, MR-PRESSO and leave-one-out analyses; 3.3 and 3.6 present sensitivity results; 4 Discussion (“The Interplay of Stress, Inflammation, and Sleep”) and Supplementary Table S1 describe exploratory MR analyses of HRV traits on CRP and IL-6. |
| 9 | **Software and pre-registration** |  |  |  |
|  | a) | Name statistical software and package(s), including version and settings used | 6 | 2.4 MR Analysis states analyses were performed in R 4.5.2 using the “TwoSampleMR” package (version 0.6.24) and related functions. |
|  | b) | State whether the study protocol and details were pre-registered (as well as when and where) | N/A | No pre-registration; not applicable. |
|  | **RESULTS** |  |  |  |
| 10 | **Descriptive data** |  |  |  |
|  | a) | Report the numbers of individuals at each stage of included studies and reasons for exclusion. Consider use of a flow diagram | 4, 7–8 | Sample sizes for all GWAS described in 2.2 Data Sources and Instruments; numbers of SNP instruments retained after selection and harmonisation are given in 3.1 Selection of IVs for MR Analysis and 3.4 Selection of IVs for Reverse MR Analysis. |
|  | b) | Report summary statistics for phenotypic exposure(s), outcome(s), and other relevant variables (e.g. means, SDs, proportions) | 4, 7–8 | HRV traits and insomnia phenotype definitions and sample characteristics described in 2.2; distributions of instruments and main MR estimates summarised in 3.2 MR Results for HRV and Insomnia and 3.5 Reverse MR Analysis of Insomnia and HRV. |
|  | c) | If the data sources include meta-analyses of previous studies, provide the assessments of heterogeneity across these studies | N/A | Not applicable; primary GWAS (Nolte et al., FinnGen) used directly via summary statistics. |
|  | d) | For two-sample MR:  i.  Provide justification of the similarity of the genetic variant-exposure associations between the exposure and outcome samples  ii.  Provide information on the number of individuals who overlap between the exposure and outcome studies | 4 | 2.2 Data Sources and Instruments notes that all participants are of European ancestry and data come from independent consortia (IEU Open GWAS vs FinnGen), implying minimal sample overlap. |
| 11 | **Main results** |  |  |  |
|  | a) | Report the associations between genetic variant and exposure, and between genetic variant and outcome, preferably on an interpretable scale | 7-8 | 3.2 MR Results for HRV and Insomnia and 3.5 Reverse MR Analysis of Insomnia and HRV plus Tables 1–4 present ORs, 95% CIs and p-values; scatter plots and forest/funnel plots described as Supplementary Figures S1–S4. |
|  | b) | Report MR estimates of the relationship between exposure and outcome, and the measures of uncertainty from the MR analysis, on an interpretable scale, such as odds ratio or relative risk per SD difference | 7-8 | 3.2 MR Results for HRV and Insomnia and 3.5 Reverse MR Analysis of Insomnia and HRV plus Tables 1–4 present ORs, 95% CIs and p-values; scatter plots and forest/funnel plots described as Supplementary Figures S1–S4. |
|  | c) | If relevant, consider translating estimates of relative risk into absolute risk for a meaningful time period | 7-8 | 3.2 MR Results for HRV and Insomnia and 3.5 Reverse MR Analysis of Insomnia and HRV plus Tables 1–4 present ORs, 95% CIs and p-values; scatter plots and forest/funnel plots described as Supplementary Figures S1–S4. |
|  | d) | Consider plots to visualize results (e.g. forest plot, scatterplot of associations between genetic variants and outcome versus between genetic variants and exposure) | 7-8 | 3.2 MR Results for HRV and Insomnia and 3.5 Reverse MR Analysis of Insomnia and HRV plus Tables 1–4 present ORs, 95% CIs and p-values; scatter plots and forest/funnel plots described as Supplementary Figures S1–S4. |
| 12 | **Assessment of assumptions** |  |  |  |
|  | a) | Report the assessment of the validity of the assumptions | 7-8 | 3.3 Sensitivity Analysis of MR Results for HRV and Insomnia and 3.6 Sensitivity Analysis of Insomnia on HRV report heterogeneity (Cochran’s Q), MR-Egger intercepts and MR-PRESSO global tests. |
|  | b) | Report any additional statistics (e.g., assessments of heterogeneity across genetic variants, such as *I^2^*, Q statistic or E-value) | 7-8 | 3.3 Sensitivity Analysis of MR Results for HRV and Insomnia and 3.6 Sensitivity Analysis of Insomnia on HRV report heterogeneity (Cochran’s Q), MR-Egger intercepts and MR-PRESSO global tests. |
| 13 | **Sensitivity analyses and additional analyses** |  |  |  |
|  | a) | Report any sensitivity analyses to assess the robustness of the main results to violations of the assumptions | 7–8, 10 | Forward and reverse MR sensitivity analyses reported in *3.3* and *3.6* with leave-one-out and funnel plots (Supplementary Figures S2–S4); exploratory CRP/IL-6 MR analyses and their interpretation discussed in *4 Discussion* (“The Interplay of Stress, Inflammation, and Sleep”) and Supplementary Table S1. No non-MR analyses conducted. |
|  | b) | Report results from other sensitivity analyses or additional analyses | 7–8, 10 | Forward and reverse MR sensitivity analyses reported in *3.3* and *3.6* with leave-one-out and funnel plots (Supplementary Figures S2–S4); exploratory CRP/IL-6 MR analyses and their interpretation discussed in *4 Discussion* (“The Interplay of Stress, Inflammation, and Sleep”) and Supplementary Table S1. No non-MR analyses conducted. |
|  | c) | Report any assessment of direction of causal relationship (e.g., bidirectional MR) | 7–8, 10 | Forward and reverse MR sensitivity analyses reported in *3.3* and *3.6* with leave-one-out and funnel plots (Supplementary Figures S2–S4); exploratory CRP/IL-6 MR analyses and their interpretation discussed in *4 Discussion* (“The Interplay of Stress, Inflammation, and Sleep”) and Supplementary Table S1. No non-MR analyses conducted. |
|  | d) | When relevant, report and compare with estimates from non-MR analyses | 7–8, 10 | Forward and reverse MR sensitivity analyses reported in *3.3* and *3.6* with leave-one-out and funnel plots (Supplementary Figures S2–S4); exploratory CRP/IL-6 MR analyses and their interpretation discussed in *4 Discussion* (“The Interplay of Stress, Inflammation, and Sleep”) and Supplementary Table S1. No non-MR analyses conducted. |
|  | e) | Consider additional plots to visualize results (e.g., leave-one-out analyses) | 7–8, 10 | Forward and reverse MR sensitivity analyses reported in *3.3* and *3.6* with leave-one-out and funnel plots (Supplementary Figures S2–S4); exploratory CRP/IL-6 MR analyses and their interpretation discussed in *4 Discussion* (“The Interplay of Stress, Inflammation, and Sleep”) and Supplementary Table S1. No non-MR analyses conducted. |
|  | **DISCUSSION** |  |  |  |
| 14 | **Key results** | Summarize key results with reference to study objectives | 9 | First paragraph of 4 Discussion summarizes bidirectional MR findings: HRV traits (pvRSA/HF, SDNN) increase insomnia risk, while insomnia shows no causal effect on HRV. |
| 15 | **Limitations** | Discuss limitations of the study, taking into account the validity of the IV assumptions, other sources of potential bias, and imprecision. Discuss both direction and magnitude of any potential bias and any efforts to address them | 11-12 | Methodological Advantages and Limitations subsection discusses ancestry restriction, FinnGen sample size and phenotype definition, residual pleiotropy, and the lifelong-effect nature of MR estimates. |
| 16 | **Interpretation** |  |  |  |
|  | a) | Meaning: Give a cautious overall interpretation of results in the context of their limitations and in comparison with other studies | 9–12 | Discussion integrates MR results with existing literature on insomnia and HRV, emphasising that HRV dysregulation may contribute to insomnia and highlighting need for complementary evidence from trials and mechanistic studies. |
|  | b) | Mechanism: Discuss underlying biological mechanisms that could drive a potential causal relationship between the investigated exposure and the outcome, and whether the gene-environment equivalence assumption is reasonable. Use causal language carefully, clarifying that IV estimates may provide causal effects only under certain assumptions | 9–10 | Subsections Heart Rate Variability and the Autonomic Nervous System and The Interplay of Stress, Inflammation, and Sleep discuss SNS/PNS imbalance, stress, inflammation and HRV, and interpret MR findings within this mechanistic framework using cautious causal language. |
|  | c) | Clinical relevance: Discuss whether the results have clinical or public policy relevance, and to what extent they inform effect sizes of possible interventions | 12 | Future Directions discusses HRV-targeted non-pharmacological therapies (e.g. CBT-I, biofeedback, acupuncture, taVNS), potential for HRV monitoring via wearables, and implications for insomnia prevention and management. |
| 17 | **Generalizability** | Discuss the generalizability of the study results (a) to other populations, (b) across other exposure periods/timings, and (c) across other levels of exposure | 11-12 | Methodological Advantages and Limitations and Future Directions note restriction to European ancestry, limitations of current GWAS phenotypes, and the need to test findings in other populations and clinical subgroups. |
|  | **OTHER INFORMATION** |  |  |  |
| 18 | **Funding** | Describe sources of funding and the role of funders in the present study and, if applicable, sources of funding for the databases and original study or studies on which the present study is based | 13 | Funding section lists supporting agencies and funding numbers and notes their roles. |
| 19 | **Data and data sharing** | Provide the data used to perform all analyses or report where and how the data can be accessed, and reference these sources in the article. Provide the statistical code needed to reproduce the results in the article, or report whether the code is publicly accessible and if so, where | 6-7 | 2.2 Data Sources and Instruments and 2.4 MR Analysis reference IEU Open GWAS and FinnGen URLs and packages used (TwoSampleMR); all data are from open GWAS resources and analyses are based on standard, publicly available code. |
| 20 | **Conflicts of Interest** | All authors should declare all potential conflicts of interest | 13 | Acknowledgments / Conflicts of Interest statement declares that the authors report no conflicts of interest. |
