# Supplementary material for: What comes first: Heart rate variability changes or insomnia? A causal investigation using Mendelian randomization
Source: Int J Clin Health Psychol. 2025 Dec 12;25(4):100656. doi: 10.1016/j.ijchp.2025.100656 (PMC12765190; doi:10.1016/j.ijchp.2025.100656)
Supplement: Supplementary file 4 [file mmc4.pdf]

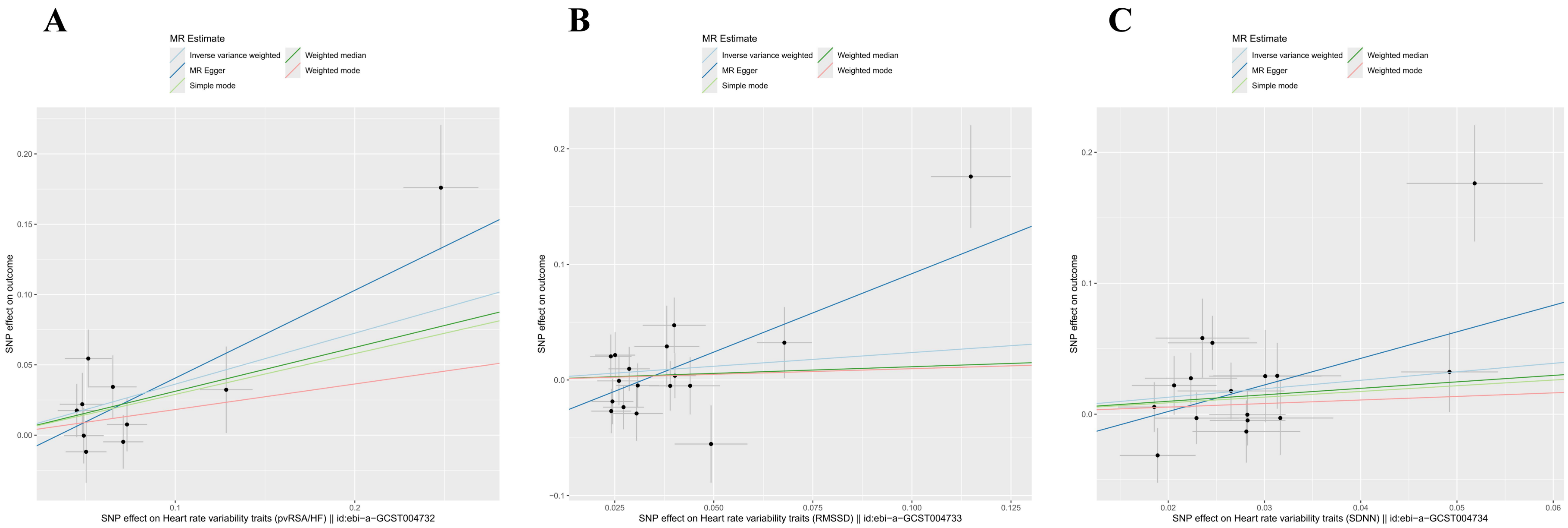

Figure S1. Scatter plots depicting MR results for HRV and insomnia.

Panels A, B, and C correspond to the HRV datasets for pvRSA/HF, RMSSD and SDNN, respectively. MR, Mendelian randomization; pvRSA/HF, the peak-valley respiratory sinus arrhythmia or high-frequency power; RMSSD, the root mean square of successive RR interval differences; SDNN, the standard deviation of normal-to-normal intervals; SNP, single nucleotide polymorphism.
