# Supplementary material for: What comes first: Heart rate variability changes or insomnia? A causal investigation using Mendelian randomization
Source: Int J Clin Health Psychol. 2025 Dec 12;25(4):100656. doi: 10.1016/j.ijchp.2025.100656 (PMC12765190; doi:10.1016/j.ijchp.2025.100656)
Supplement: Supplementary file 5 [file mmc5.pdf]

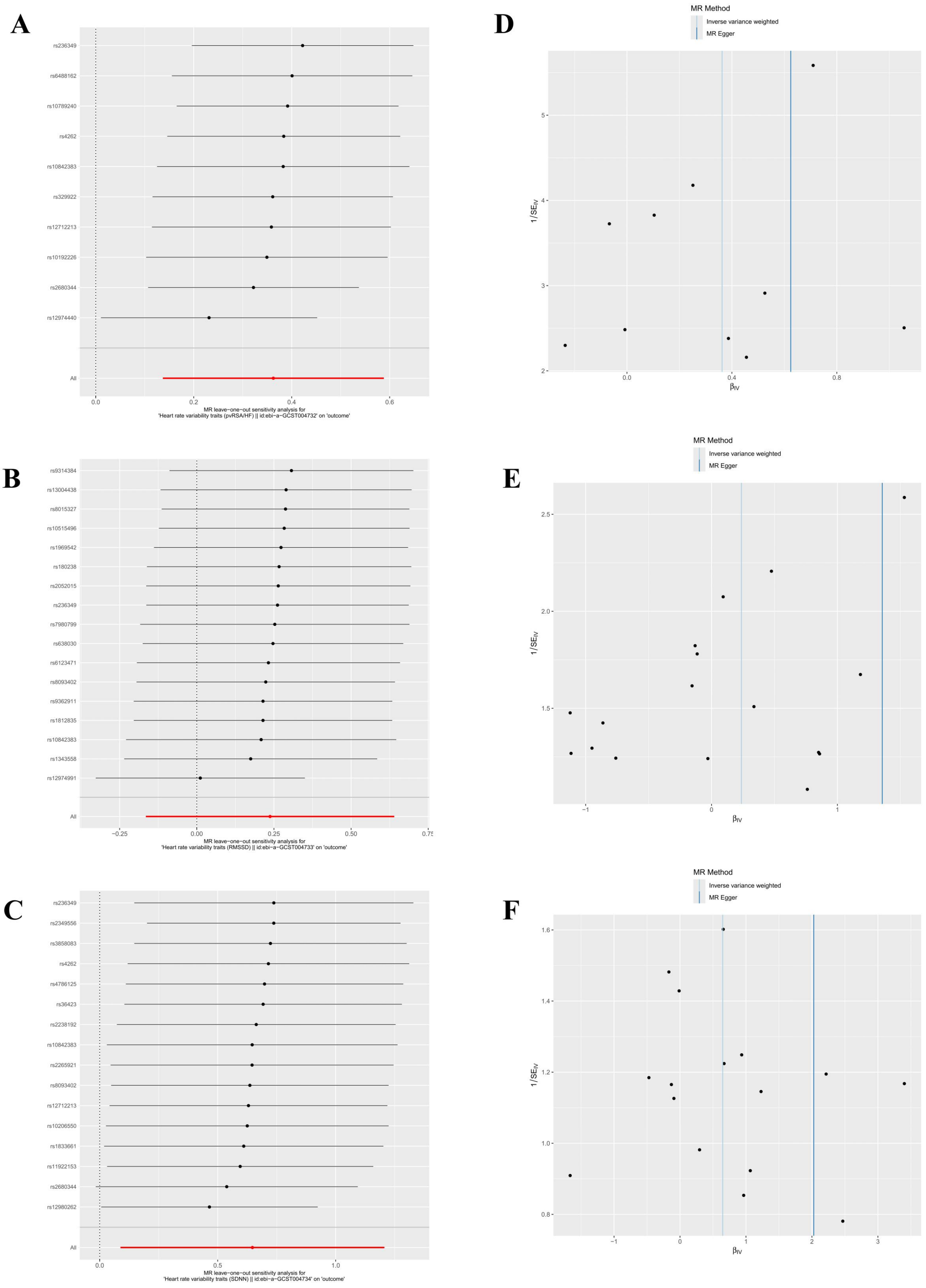

Figure S2. Leave-one-out validation and funnel plots for MR of HRV and insomnia.

This figure presents validation and bias-assessment analyses for the MR study of HRV traits and insomnia. **Panels S2A–S2C** show leave-one-out validation plots for

the HRV datasets (pvRSA/HF, RMSSD, and SDNN, respectively), assessing the influence of excluding individual genetic variants on the IVW causal estimates (red line, overall IVW estimate). **Panels S2D–S2F** display the corresponding funnel plots for the same datasets, evaluating asymmetry and heterogeneity in the MR analyses.

Abbreviations: MR, Mendelian randomization; HRV, heart rate variability; pvRSA/HF, peak-valley respiratory sinus arrhythmia or high-frequency power; RMSSD, root mean square of successive RR interval differences; SDNN, standard deviation of normal-to-normal intervals; SNP, single nucleotide polymorphism.
