# Supplementary material for: What comes first: Heart rate variability changes or insomnia? A causal investigation using Mendelian randomization
Source: Int J Clin Health Psychol. 2025 Dec 12;25(4):100656. doi: 10.1016/j.ijchp.2025.100656 (PMC12765190; doi:10.1016/j.ijchp.2025.100656)
Supplement: Supplementary file 8 [file mmc8.docx]

**Supplementary Table S1.** Exploratory two-sample Mendelian randomization estimates for the causal effects of heart rate variability (HRV) traits on circulating inflammatory markers (IVW method).

| **Exposure**  **(HRV trait)** | **Outcome**  **(inflammatory marker)** | **nsnp** | **Beta** | **SE** | **OR** | **95% CI for OR** | **p-value** |
| --- | --- | --- | --- | --- | --- | --- | --- |
| pvRSA/HF | C-reactive protein (CRP) | 5 | -0.0196 | 0.0233 | 0.98 | 0.94–1.03 | 0.409 |
| SDNN | C-reactive protein (CRP) | 6 | -0.0571 | 0.0958 | 0.94 | 0.78–1.14 | 0.551 |
| pvRSA/HF | Interleukin-6 (IL-6) | 5 | -0.2143 | 0.2003 | 0.81 | 0.55–1.20 | 0.285 |
| SDNN | Interleukin-6 (IL-6) | 6 | -0.0423 | 0.5446 | 0.96 | 0.33–2.79 | 0.938 |

***Abbreviations:*** *HRV, Heart rate variability; pvRSA/HF, Peak-valley respiratory sinus arrhythmia or high-frequency power SDNN, Standard deviation of normal-to-normal intervals;* IVW, inverse variance weighted.
